# Jasmonic acid is not a biosynthetic intermediate to produce the pyrethrolone moiety in pyrethrin II

**DOI:** 10.1038/s41598-020-63026-3

**Published:** 2020-04-14

**Authors:** Ryo Matsui, Kisumi Takiguchi, Naoshige Kuwata, Katsunari Oki, Kosaku Takahashi, Kazuhiko Matsuda, Hideyuki Matsuura

**Affiliations:** 1 Laboratory of Natural Product Chemistry, Division of Fundamental AgriScience Research, Research Faculty of Agriculture, Hokkaido University, Sapporo, 060-8589 Japan; 2 Graduate School of Agriculture, Faculty of Agriculture, Kinki University, Nakamachi, Nara, 631-8505 Japan; 3 Present Address: Department of Nutritional Science, Faculty of Applied BioScience, Tokyo University of Agriculture, 1-1-1 Sakuragaoka, Setagaya-ku, Tokyo, 156-8502 Japan

**Keywords:** Secondary metabolism, Plant ecology

## Abstract

Pyrethrum (*Tanacetum* *cinerariifolium*) produces insecticidal compounds known as pyrethrins. Pyrethrins are esters; the acid moiety is either *trans*-chrysanthemic acid or pyrethric acid and the alcohol moiety of pyrethrins is either pyrethrolone, cinerolone, or jasmolone. It was generally accepted that *cis*-jasmone was biosynthetic intermediate to produce the alcohol moieties of pyrethrin, and the biosynthetic origin of the *ci*s-jasmone was postulated to be jasmonic acid. However, there was no direct evidence to prove this hypothesis. In order to uncover the origin of pyrethrolone moiety in pyrethrin II, feeding experiments were performed employing deuterium- and ^13^C-labeled compounds as substrates, and the expected labeled compounds were analyzed using UPLC MS/MS system. It was found that the pyrethrolone moiety in pyrethrin II was derived from 12-oxo-phytodienoic acid (OPDA), *iso*-OPDA and *cis*-jasmone but not from methyl jasmonate and 3-oxo-2-(2′-[*Z*]-pentenyl)-cyclopentane-1-hexanoic acid. The results supported that the biosynthesis of the pyrethrolone moiety in pyrethrin II partially used part of the jasmonic acid biosynthetic pathway, but not whole.

## Introduction

**Figure 1:**
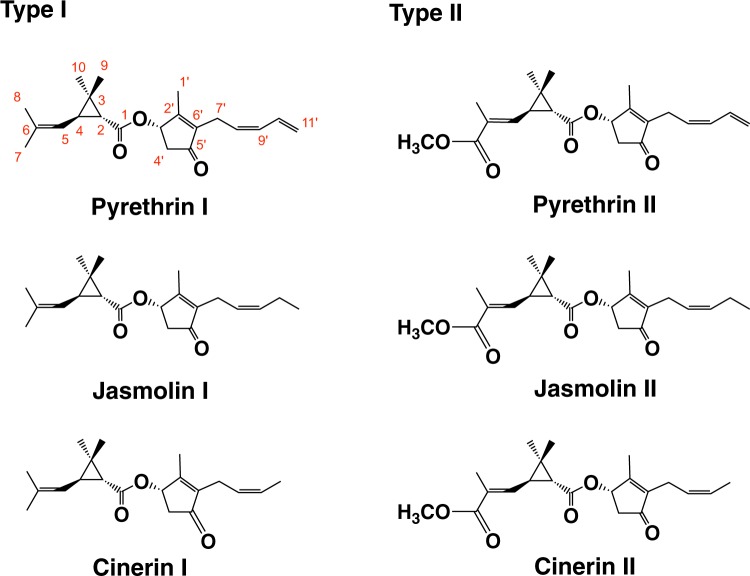
Chemical structures of pyrethrins.

**Figure 2:**
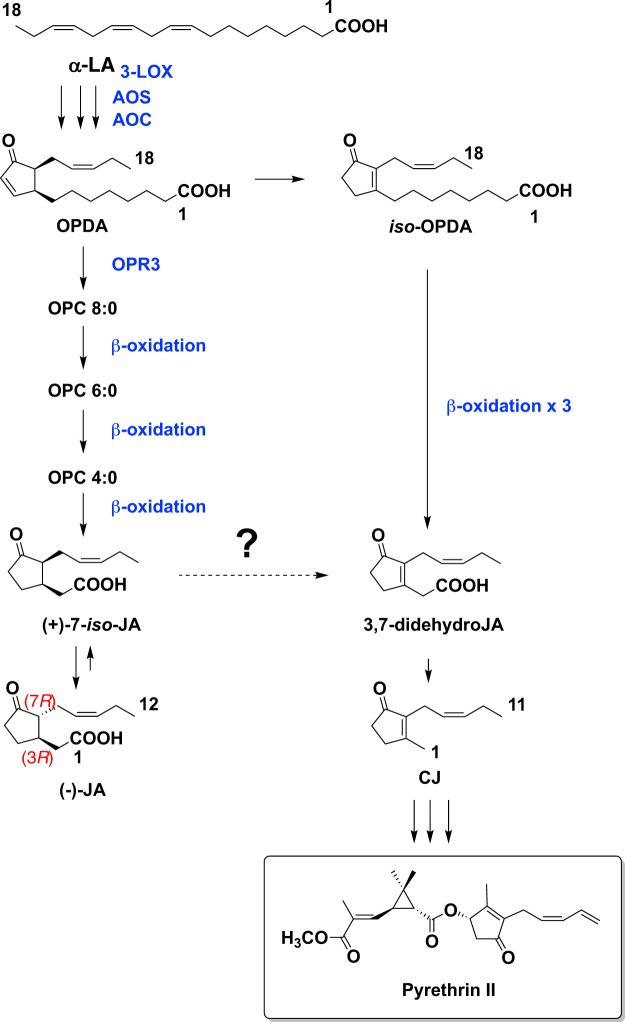
Proposed biosynthetic pathway of pyrethrin II. LOX, lipoxygenase; AOS, allene oxide synthase; AOC, allene oxide cyclase; OPR3, 12-oxo-phytodienoic acid reductase; α-LA, α-linolenic acid; OPDA, 12-oxo-phytodienoic acid; OPC 8:0, 3-oxo-2-(2′-[*Z*]-pentenyl)-cyclopentan-1-octanoic acid; OPC 6:0, 3-oxo-2-(2′-[*Z*]-pentenyl)-cyclopentane-1-hexanoic acid; OPC 4:0, 3-oxo-2-(2′-[*Z*]-pentenyl)-cyclopentane-1-butanoic acid; JA, jasmonic acid.

Pyrethrum (*Tanacetum cinerariifolium*, formerly *Chrysanthemum cinerariifolium*) is a plant in the Asteraceae family that synthesizes insecticides known as pyrethrin (Fig. 1), and it was found that the highest levels of production were observed in flowers^1,2^, whereas a smaller amount was observed in leaves. Since pyrethrin shows remarkable toxicity against a wide range of insect species but low toxicity to warm-blooded animals, pyrethrin has been used for pest control^1^. Until the middle of the 20^th^ century, Japan was the world’s largest producer of pyrethrum, and it had been thought to be ideal to collect flowers before they were fully opened. However, pyrethroids, which are synthetic analogs of pyrethrin, are used at much higher amounts than pyrethrin as pest control agents due to their lower cost to produce and their effectiveness, even though they are less safe to vertebrates and less biodegradable^3,4^. Recently, some researchers have noted the emergence of resistance among insects and toxicity toward mammals and fish^5^. From the chemical structural viewpoint, pyrethrin has ester linkages in the chemical structure and is classified into two types (Fig. 1): type I esters have a chrysanthemoyl moiety, whereas type II esters have a pyrethroyl moiety having additional ester linkage composing methoxycarbonyl group. A common and distinctive structural feature of the acid moieties is a cyclopropane ring moiety. The alcohol-containing parts of pyrethrin are either pyrethrolone, cinerolone, or jasmolone, which all have a similar basic structure with that of *cis*-jasmone. The enzyme producing the ester linkage was identified as a GDSL lipase-like protein^6^, which was encoded on *TcGLIP* (Fig. S1). The synthesis of the acid and alcohol moieties of pyrethrin is not fully understood. For the acid moiety, it was demonstrated that *T. cinerariifolium* flowers have the enzyme *trans*-chrysanthemyl diphosphate synthase (CDS), which conjugates two dimethyl allyl diphosphate (DMAPP) molecules via irregular linkage to produce *trans*-chrysanthemyl diphosphate (CDP)^7^. The identities of the enzymes responsible for the subsequent steps to produce chrysanthemic acid had been not known for long, although Xu *et al*.^8^ reported that *TcADH2* encodes an enzyme that oxidizes *trans*-chrysanthemol to *trans*-chrysanthemal and *TcALDH1* encodes an enzyme that oxidizes the aldehyde into *trans*-chrysanthemic acid (Fig. S1)^8^. With respect to the alcohol moiety, it was thought that pyrethrolone, cinerolone, and jasmolone were synthesized from *cis*-jasmone (Fig. S1), and recently, Li *et al*. found that the treatment of flower buds with *cis*-jasmone resulted in a higher level of accumulation of jasmolone, pyrethrolone, jasmolin I, and pyrethrin I than those of untreated flower buds, and also reported that *cis*-jasmone hydroxylase (CYP71AT148: TcJMH) was involved in producing jasmolone (Fig. S1)^9^. *cis*-Jasmone (Fig. S1) in plants is thought to be derived from jasmonic acid (JA) based on the feeding experiment of [^2^H_1_–7, ^2^H_2_–5, ^2^H_2_–2] JA to *Jasrninurn rincospernurn*, resulting into giving [^2^H_2_–4, ^2^H_2_–1] *cis*-jasmone, (Fig. S2a)^10^. JA is a phytohormone and a pivotal inducer of plant wound responses to insects and necrotrophic pathogens^11–13^. The biosynthesis of JA starts with the oxygenation of α-linolenic acid (LA) in the chloroplast to give (+)-7-*iso*-JA in the peroxisome^14^ via OPDA, 3-oxo-2-(2′-[*Z*]-pentenyl)-cyclopentan-1-octanoic acid (OPC 8:0), 3-oxo-2-(2′-[*Z*]-pentenyl)-cyclopentane-1-hexanoic acid (OPC 6:0), and 3-oxo-2-(2′-[*Z*]-pentenyl)-cyclopentane-1-butanoic acid (OPC 4:0) (Fig. 2). In general, (+)-7-*iso*-JA is readily epimerized to afford (−)-JA having an absolute configuration of (3 *R*,7 *R*) (Fig. 2) during isolation procedure. The synthesized (+)-7-*iso*-JA is metabolized to give other jasmonates, including methyl ester, 12-OH oxidized and 12-*O*-glucopyranosylated forms, and JA-amino acid conjugates such as (+)-7-*iso*-jasmonoyl-L-isoleucine. It is generally accepted that (+)-7-*iso*-jasmonoyl-L-isoleucine is true biological active form of JA signaling and (−)-7-*iso*-jasmonoyl-L-isoleucine was the compound obtained by isomerization during isolation procedure. As mentioned above, the biosynthetic pathway producing *cis*-jasmone was postulated in the report of Koch *et al*. (Fig. S2a)^10^, in which JA was involved as an intermediate to afford *cis*-jasmone. It was well known that JA accumulation was enhanced by wounding, and Loughrin *et al*.^15^ reported that *cis*-jasmone was also induced by wounding. Since pyrethrin biosynthesis in leaves was also accelerated by wounding, it was suggested that the synthesis of the alcohol moieties of pyrethrins may be tied to the JA biosynthetic pathway^16,17^, in which JA was thought as an intermediate to produce *cis*-jasmone. However, Dabrowska *et al*. reported another biosynthetic pathway of *cis*-jasmone via *iso*-OPDA (Fig. 2)^18^, and it was recently found that *cis*-jasmone produced in the pathogenic fungus, *Lasiodiplodia theobromae* was derived from α-LA, OPDA, and *iso*-OPDA but not from OPCs 8:0, 6:0, 4:0 and JA^19,20^. Therefore, at present, it cannot be clearly stated that the biosynthetic origin of pyrethrolone, cinerolone, and jasmolone moieties in pyrethrin is JA, although it was generally accepted that JA is involved to produce the alcohol moieties in pyrethrin. In this report, based on experiments using deuterium- and ^13^C-labeled compounds, it was revealed that the pyrethrolone moiety in pyrethrin II was derived from OPDA, *iso*-OPDA and *cis*-jasmone but not from JA.

## Results

### Analysis of pyrethrin in the leaves of pyrethrum (*Tanacetum cinerariifolium*) using UPLC MS/MS

Xu *et al*.^8^ and Li *et al*.^9^ employed GC-MS method to detect the target compounds, and it was reported that the endogenous amount of pyrethrins in leaves was much lower than that in blossoming flowers. However, in this study, the analyses were performed using UPLC MS/MS in MRM mode due to the shorter time for measurements and its higher detection performance. Authentic samples of pyrethrins I and II and jasmolin I were used to establish optimum conditions for the measurement (Table S1, Fig. S3), and the parameters for measuring cinerins I and II and jasmolin II were set based on those of pyrethrins I and II and jasmolin I (Table S1). A whole plant extract of pyrethrum was used to detect pyrethrins I and II, jasmolins I and II, and cinerins I and II, and it was found that endogenous amounts of the target compounds were high enough to be detected using the established conditions, in which the peak having highest value of area was that of pyrethrin II (Fig. S4). Because it was found that the major pyrethrin in whole plant extract was pyrethrin II, and the compound was able to be detected sufficiently even using with the small amount of the leaf, we planned to evaluate the biosynthetic origin of the pyrethrolone moiety in pyrethrin II.

### Airborne [^2^H_3_–12, ^2^H_2_–11, ^2^H_1_–10] (±)-MeJA was incorporated into JA, 12-OH-JA, and JA-Ile but not into pyrethrin II

**Figure 3:**
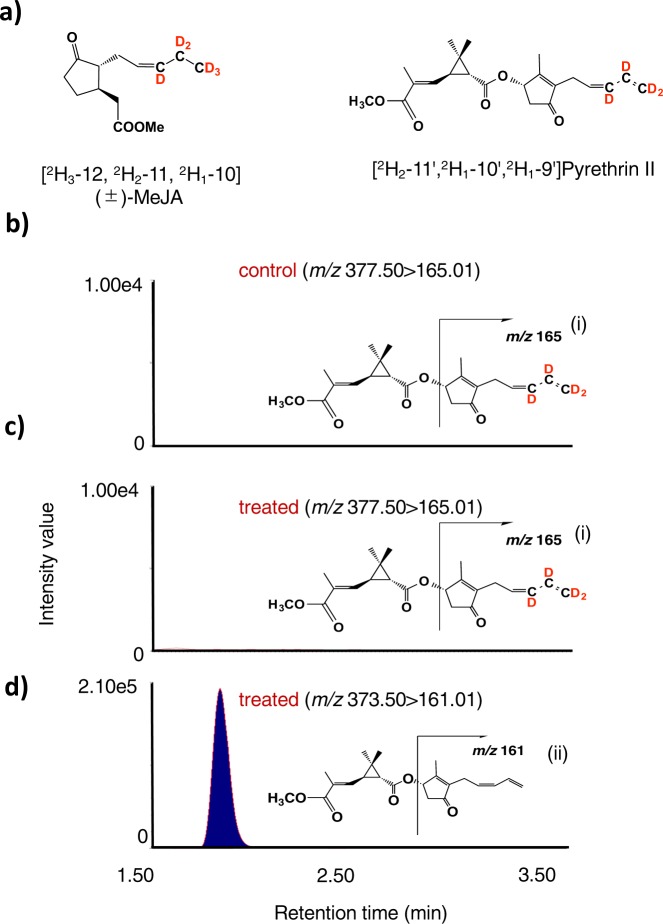
Feeding experiment using deuterium labeled (±)-MeJA. (**a**) Chemical structure of [^2^H_3_–12, ^2^H_2_–11, ^2^H_1_–10](±)-MeJA and pyrethrin II having expected labeled pattern. (**b**) UPLC MS/MS chromatogram of the leaf extract of untreated plants (control) detecting for pyrethrin II with the expected labeled pattern. (**c**) UPLC MS/MS chromatogram of the leaf extract of treated plants detecting for pyrethrin II having the expected labeled pattern. (**d**) UPLC MS/MS chromatogram of the leaf extract of treated plants detecting for endogenous pyrethrin II. Insets indicate the fragmentation patterns of deuterium-labeled pyrethrin II (i) and endogenous pyrethrin II (ii).

Synthesis of [^2^H_3_–12, ^2^H_2_–11, ^2^H_1_–10] (±)-JA (JA-*d*_6_) was performed according to the reported method^21^, and the obtained compound was treated with CH_2_N_2_ in Et_2_O to give [^2^H_3_–12, ^2^H_2_–11, ^2^H_1_–10] (±)-MeJA (Fig. 3a, (±)-MeJA-*d*_6_). In the case of the applied compound being incorporated into JA and JA derivatives such as 12-OH-JA and JA-L-Ile, the predicted labeled compounds were [^2^H_3_–12,^2^H_2_–11,^2^H_1_–10] JA, [^2^H_2_–12,^2^H_2_–11,^2^H_1_–10], 12-OH-JA, and [^2^H_3_–12,^2^H_2_–11,^2^H_1_–10] JA-L-Ile. Chromatograms of UPLC MS/MS for authentic JA-L-Ile, JA, and 12-OH-JA are given in Fig. S5. On the other hand, in the case of the applied compound being incorporated into pyrethrin II, the predicted labeled compound was [^2^H_2_–11′,^2^H_1_–10′,^2^H_1_–9′] pyrethrin II (Fig. 3a). Pyrethrum plants that grew in a greenhouse for 35 days were placed in a semi-closed container with filter paper loaded with (±)-MeJA-*d*_6_ (1 mg × 3) for six plants, and after 24 h, the upper parts of the plants were harvested and extracted. To survey the presence of the predicted deuterium-labeled and the naturally occurring compounds, UPLC-MS/MS analysis was performed according to the condition given in Material and Methods. As described in previous reports^22,23^, the airborne deuterium-labeled MeJA were metabolized into JA, 12-OH-JA, and JA-Ile (Fig. S6). Based on the comparisons of the peak area ratio (deuterium-labeled compounds/naturally occurring compounds) for JA, 12-OH-JA, and JA-Ile, it was found that the labeled compounds exhibited greater accumulation than that of the naturally occurring compounds. However, the expected transition ion peak ascribed for labeled pyrethrin II was not detected in the chromatographs analyzing the extracts derived from either treated or untreated plants, even though naturally occurring pyrethrin II was detected in the extract of the treated plants (Fig. 3).

### [^2^H_3_–16,^2^H_2_–15,^2^H_1_–14] (±)-OPC 6:0 applied on leaves was incorporated into 12-OH-JA but not into pyrethrin II

The synthesis of [^2^H_3_–16,^2^H_2_–15,^2^H_1_–14] (±)-OPC 6:0 ((±)-OPC 6:0-*d*_6_, Fig. S7a) was performed according to the reported method^24^. An anhydrous lanolin paste (100 mg) containing (±)-OPC 6:0-*d*_6_ (10 μmol) was applied to the leaves of pyrethrum, and the treated leaves were harvested after 24 h. UPLC MS/MS analysis in MRM mode revealed that the applied (±)-OPC 6:0-*d*_6_ was incorporated into [^2^H_2_–12,^2^H_2_–11,^2^H_1_–10] 12-OH-JA in the treated plant (Fig. S7b). However, the expected transition ion peak ascribed for labeled pyrethrin II (Fig. S7a) was not detected in the chromatographs analyzing extract derived from either treated or untreated plants, even though naturally occurring pyrethrin II was detected in the extract of the treated plants (Fig. S7c).

### [^2^H_2_–4, ^2^H_2_–3, ^2^H_3_–1] *cis*-jasmone applied on leaves with lanolin paste was incorporated into pyrethrin II

**Figure 4:**
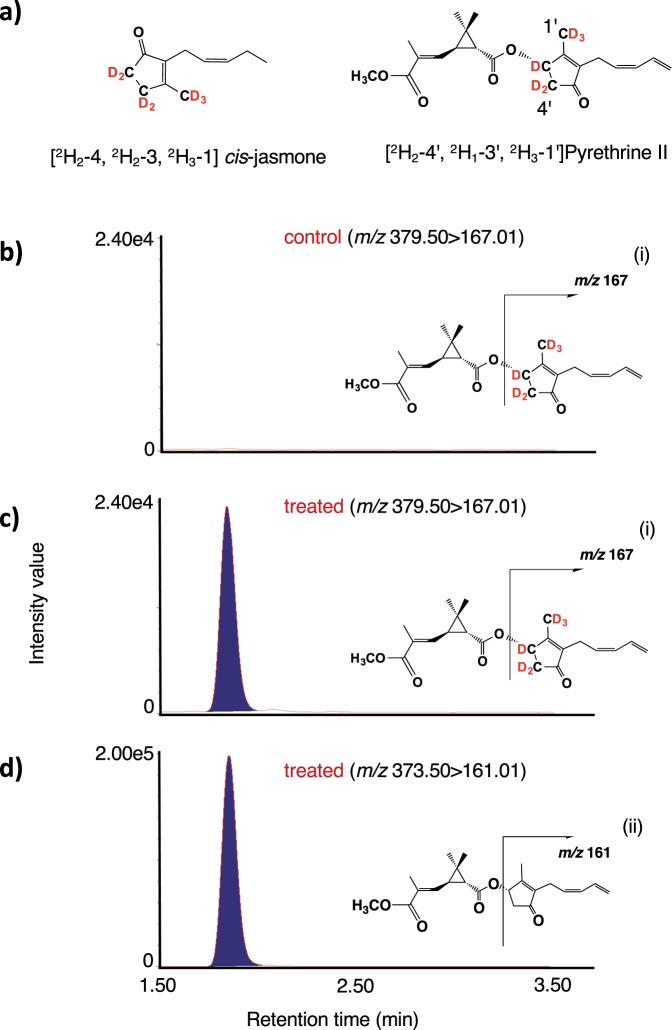
Feeding experiment using deuterium-labeled *cis*-jasmone. (**a**) Chemical structure of [^2^H_2_–4, ^2^H_2_–3, ^2^H_3_–1] *cis*-jasmone and [^2^H_2_–4′, ^2^H_1_–3′, ^2^H_3_–1′] pyrethrin II. (**b**) UPLC MS/MS chromatogram of the leaf extract of untreated plants (control) detecting for pyrethrin II with the expected labeled pattern. (**c**) UPLC MS/MS chromatogram of the leaf extract of treated plants detecting for pyrethrin II having the expected labeled pattern. (**d**) UPLC MS/MS chromatogram of the leaf extract of treated plants detecting for endogenous pyrethrin II. Insets indicate the fragmentation patterns of deuterium labeled pyrethrin II (i) and pyrethrin II (ii).

As mentioned above, it was found that the treatment of flower buds with *cis*-jasmone resulted in higher levels of accumulation of jasmolone, pyrethrolone, jasmolin I, and pyrethrin I than those of untreated flower buds, and it was concluded that the applied compound was incorporated^9^. However, the incorporation of the applied compound was not completely proven due to the use of unlabeled *cis*-jasmone, which made it impossible to distinguish between the exogenously applied compound and the plant-produced compound. The method employed by Li *et al*.^9^ could not exclude the possibility that exogenously applied *cis*-jasmone only accelerated the biosynthesis of pyrethrins. Synthesis of [^2^H_2_–4, ^2^H_2_–3, ^2^H_3_–1] *cis*-jasmone (Fig. 4a, CJ-*d*_7_) was performed according to the reported method^19^, and used for feeding experiments. In the case of the compound being incorporated into pyrethrin II, the predicted labeled compound was [^2^H_2_–4′, ^2^H_1_–3′, ^2^H_3_–1′] pyrethrin II (Fig. 4a). An anhydrous lanolin paste (100 mg) containing CJ-*d*_7_ (10 μmol) was applied to the leaves of pyrethrum, and the treated leaves were harvested after 24 h. The analyses using UPLC MS/MS in MRM mode were performed for the detection of [^2^H_2_–4′, ^2^H_1_–3′, ^2^H_3_–1′] and endogenous pyrethrin II in untreated and treated plants, and the chromatograms are given in Fig. 4, which revealed that the exogenously applied labeled compound was incorporated into pyrethrin II in treated plants (Fig. 4c), and the labeled compounds were not detected in the untreated plants (Fig. 4b). Based on the comparisons of the peak area ratio (deuterium labeled compounds/naturally occurring compounds) for pyrethrin II, it was found that the labeled compounds accumulated to approximately one tenth of the amount of accumulated naturally occurring compounds (Fig. 4d).

### [^2^H_1_–16,^2^H_1_–15,^2^H_2_–11, ^2^H_2_–10, ^2^H_2_–8, ^2^H_2_–2] Methyl *iso*-OPDA applied on leaves was incorporated into pyrethrin II

**Figure 5:**
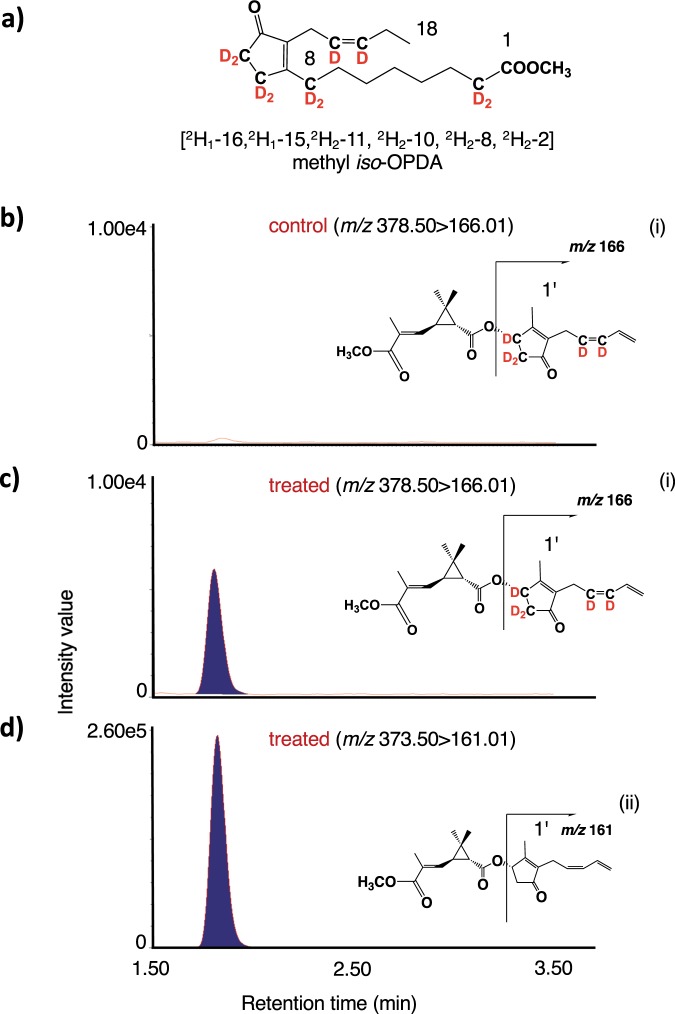
Feeding experiment using deuterium-labeled methyl *iso*-OPDA. (**a**) Chemical structure of [^2^H_1_–16, ^2^H_1_–15, ^2^H_2_–11, ^2^H_2_–10, ^2^H_2_–8, ^2^H_2_–2] methyl *iso*-OPDA. (**b**) UPLC MS/MS chromatogram of the leaf extract of untreated plants (control) detecting for pyrethrin II with the expected labeled pattern. (**c**) UPLC MS/MS chromatogram of the leaf extract of treated plants detecting for pyrethrin II having the expected labeled pattern. (**d**) UPLC MS/MS chromatogram of the leaf extract of treated plants detecting for endogenous pyrethrin II. Insets indicate the fragmentation patterns of deuterium-labeled pyrethrin II (i) and endogenous pyrethrin II (ii).

[^2^H_1_–16,^2^H_1_–15,^2^H_2_–11, ^2^H_2_–10, ^2^H_2_–8, ^2^H_2_–2] Methyl *iso*-OPDA (Fig. 5a, Me-*iso*-OPDA-*d*_10_) was synthesized according to the reported method^19^. In the case of the applied compound being incorporated into pyrethrin II, two types of labeled compounds were predicted (Fig. S8). One was [^2^H_1_–9′,^2^H_1_–8′,^2^H_2_–4′, ^2^H_1_–3′, ^2^H_2_–1′] pyrethrin II (Fig. S8a) assumed from the biosynthetic pathway reported by Koch *et al*.^10^, and the other was [^2^H_1_–9′,^2^H_1_–8′,^2^H_2_–4′, ^2^H_1_–3′] pyrethrin II (Fig. S8b) by Matsui *et al*.^19,20^. An anhydrous lanolin paste (100 mg) containing Me-*iso*-OPDA-*d*_10_ (10 μmol) was applied to the leaves of pyrethrum, and the treated leaves were harvested after 24 h. The analyses using UPLC MS/MS in MRM mode targeting endogenous, [^2^H_1_–9′,^2^H_1_–8′,^2^H_2_–4′, ^2^H_1_–3′, ^2^H_2_–1′], and [^2^H_1_–9′,^2^H_1_–8′,^2^H_2_–4′, ^2^H_1_–3′] pyrethrin II were performed. In the case of analyzing treated plants, the chromatograms for detecting [^2^H_1_–9′,^2^H_1_–8′,^2^H_2_–4′, ^2^H_1_–3′] pyrethrin II produced a peak (Fig. 5c) but [^2^H_1_–9′,^2^H_1_–8′,^2^H_2_–4′, ^2^H_1_–3′ ^2^H_2_–1′] pyrethrin II did not (Fig. S9b), which indicated that Me-*iso*-OPDA was metabolized into pyrethrolone moiety of pyrethrin II accompanying with replacements of protons at C-8 position. We also could not find the peak derived from the labeled compound in the untreated plants (Fig. 5b). Based on the comparisons of the peak area ratio (deuterium-labeled compounds/naturally occurring compounds), it was found that the labeled compounds accumulated to approximately one fiftieth of the amount of accumulated naturally occurring compounds (Fig. 5d).

### [U-^13^C] (+)-OPDA applied on leaves was incorporated into JA derivatives and pyrethrin II

**Figure 6:**
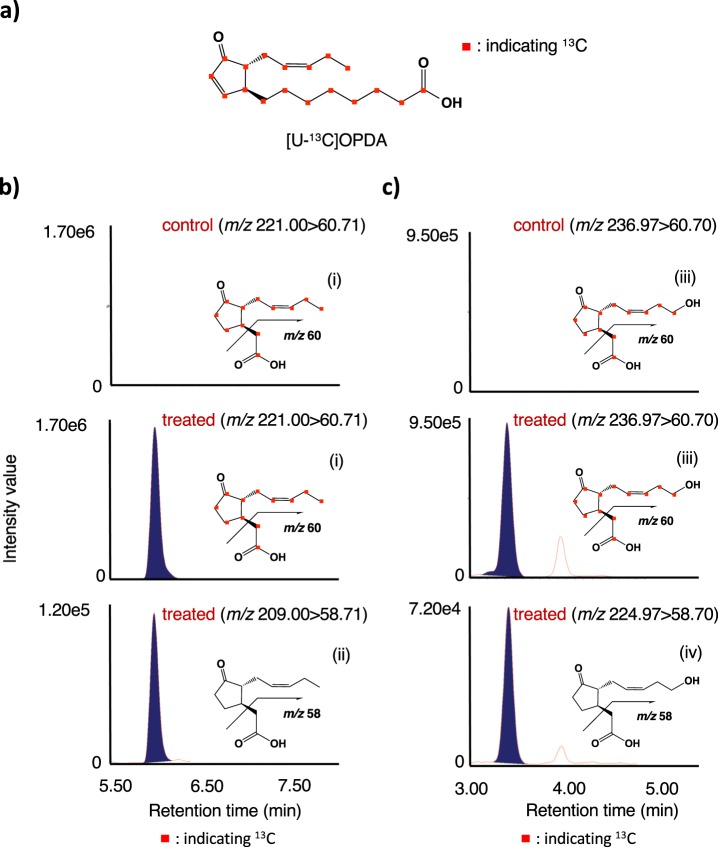
Feeding experiment using uniformly ^13^C labeled (+)-OPDA. (**a**) Chemical structure of uniformly ^13^C labeled (+)-OPDA. (**b**) UPLC MS/MS chromatograms of the leaf extract of untreated (control) and treated plants. Upper and middle panels: analyzing JA with the expected labeled pattern in the control and treated plants, respectively. Lower panel: analyzing endogenous JA in the treated plants. (**c**) UPLC MS/MS chromatograms of the leaf extract of untreated (control) and treated plants. Upper and middle panels: analyzing 12-OH-JA with the expected labeled pattern in the control and treated plants, respectively. Lower panel: analyzing endogenous 12-OH-JA in the treated plants. Insets indicate the fragmentation patterns of uniformly ^13^C labeled JA (i), endogenous JA (ii), uniformly ^13^C labeled 12-OH-JA (iii), and endogenous 12-OH-JA (iv).

**Figure 7:**
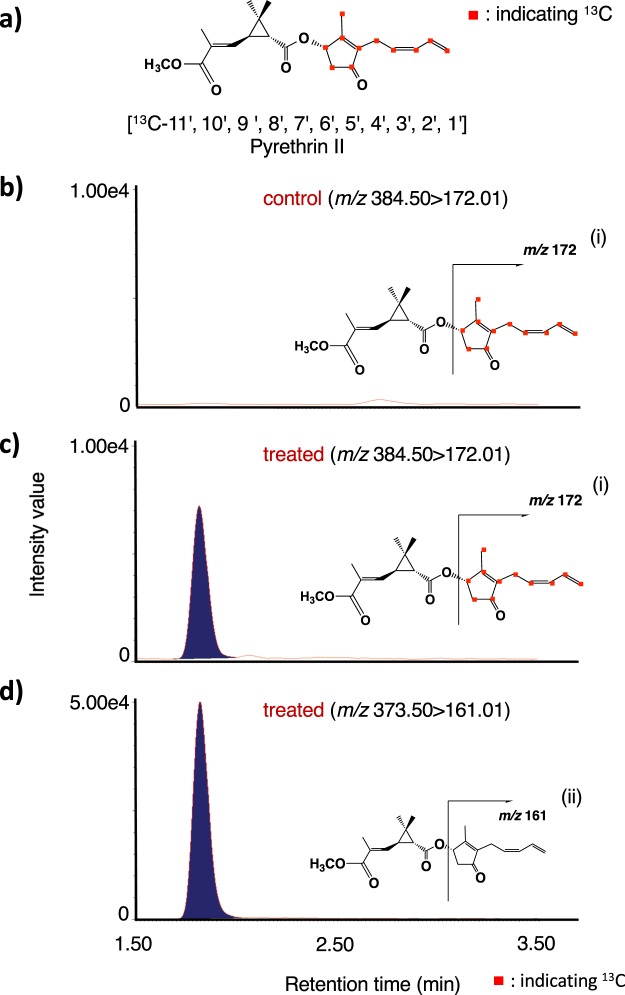
Feeding experiment using uniformly ^13^C labeled (−)-OPDA. (**a**) Chemical structure of pyrethrin II having expected ^13^C labeled pattern. (**b**) UPLC MS/MS chromatogram of the leaf extract of untreated plants (control) analyzing pyrethrin II with the expected labeled pattern. (**c**) UPLC MS/MS chromatogram of the leaf extract of treated plants with the labeled compound analyzing pyrethrin II having the expected labeled pattern. (**d**) UPLC MS/MS chromatogram of the leaf extract of treated plants with the labeled compound analyzing endogenous pyrethrin II. Insets indicate the fragmentation patterns of ^13^C labeled pyrethrin II (i) and endogenous pyrethrin II (ii).

It was thought that OPDA was a key intermediate in the production of JA and pyrethrin II since the compound was assumed to be a branching point in their syntheses, and we hypothesized that the feeding experiment of labeled OPDA would produce labeled JA, JA derivatives and pyrethrin II. The synthesis of uniformly ^13^C labeled ([U-^13^C]) (+)-OPDA (Fig. 6a) was performed according to the reported method^25^ using [U-^13^C] α-LA as a starting material. In the case of the applied compound being incorporated into JA and its derivatives such as 12-OH-JA and JA-Ile, the predicted labeled compounds were [U-^13^C] JA, [U-^13^C] 12-OH-JA, and [U-^13^C] JA-L-Ile. On the other hand, in the case of the compound being incorporated into pyrethrin II, the predicted labeled compound was [^13^C-11′,10′,9′,8′,7′,6′,5′,4′,3′,2′,1′] pyrethrin II (Fig. 7a). An anhydrous lanolin paste (100 mg) containing [U-^13^C] (+)-OPDA (10 μmol) was applied to the leaves of pyrethrum, and the treated leaves were harvested after 24 h. UPLC MS/MS analysis in MRM mode revealed that the applied [U-^13^C] (+)-OPDA was incorporated into [U-^13^C] JA, [U-^13^C] 12-OH-JA, and [U-^13^C] JA-L-Ile (Figs. 6b,c and S10c), and the results were not conflict with past reports^14^ indicating that (+)-OPDA was a biosynthetic intermediate of JA and JA derivatives. Incorporation of [U-^13^C] (+)-OPDA into pyrethrin II was examined, and the UPLC MS/MS chromatograms are given in Fig. 7. The compound having the predicted labeling pattern (Fig. 7c) and endogenous pyrethrin II (Fig. 7d) were detected in the treated plant, but the ^13^C-labeled compound was not detected in the untreated plants (Fig. 7b). The results supported that OPDA was a key intermediate to give JA, JA derivatives, and the pyrethrolone moiety of pyrethrin II. Based on the comparisons of the peak area ratio (^13^C labeled compounds/naturally occurring compounds) for pyrethrin II, it was found that the labeled compounds accumulated to approximately one fifth of the accumulated naturally occurring compounds.

## Discussion

### Detection of pyrethrins in the leaves of pyrethrum by UPLC MS/MS analysis

Pyrethrins have long been used as a powerful naturally derived herbicide, and their chemical structure has become the basic skeleton for developing the synthetic pyrethroid insecticides. However, the complete picture of the biosynthetic pathway of pyrethrin has not been clarified. In this report, we firstly set up a methodology to identify pyrethrin using UPLC MS/MS system (Fig. S4), and it was found that the major pyrethrin in the extract derived from the leaves of pyrethrum was pyrethrin II. Furthermore, the endogenous amount of pyrethrin II in the leaves of pyrethrum was high enough to detect using the extract derived from ca. 1 g fresh weight of leaves.

### Biosynthetic origin of the pyrethrolone moiety of pyrethrin II

**Figure 8:**
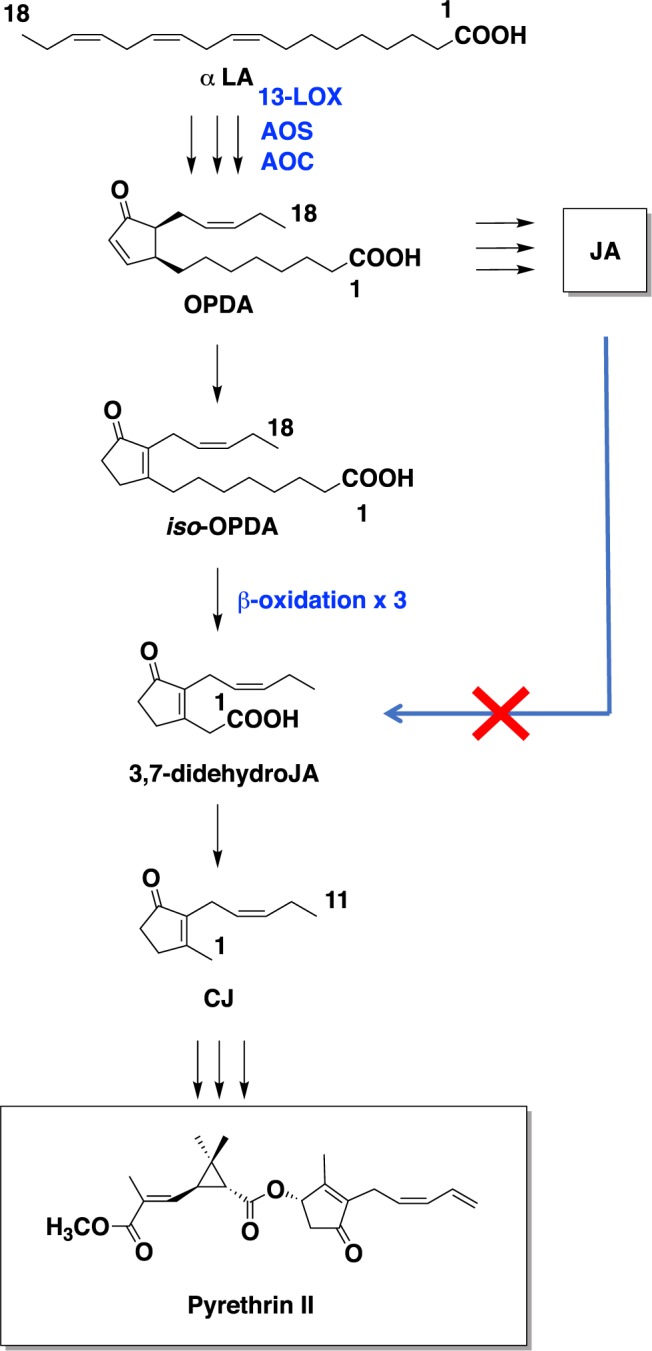
Proposed biosynthetic pathway of pyrethrolone moiety in pyrethrin II via *iso*-OPDA.

It was reported that α-LA is a starting compound to produce OPDA and the pyrethrolone moiety of pyrethrin I^14,26^. In a previous paper, we reported that a pathogenic fungus, *Lasiodiplodia theobromae*, synthesized *cis*-jasmone via *iso*-OPDA, while JA was not incorporated into the compound^19^. However, Koch *et al*. reported that exogenously applied JA was metabolized to *cis*-jasmone^10^. Thus, the biosynthetic origin of *cis*-jasmone needed to be determined. To clarify the biosynthetic origin of pyrethrolone moiety in pyrethrin II, five kinds of labeled compounds, (±)-MeJA-*d*_6_, (±)-OPC 6:0-*d*_6_, CJ-*d*_7_, Me-*iso*-OPDA-*d*_10_, and [U-^13^C] (+)-OPDA were synthesized to be used as substrates for feeding experiments. When (±)-MeJA-*d*_6_ and (±)-OPC 6:0-*d*_6_ were applied, the expected transition ion peak ascribed for labeled JAs were detected in the chromatographs (Figs. 3c,d, and S5b, S7b), although that for labeled pyrethrin II were not detected (Figs. 3b and S7c). However, CJ-*d*_7_ and Me-*iso*-OPDA-*d*_10_ were applied, the expected transition ion peak ascribed for labeled pyrethrin II were detected in the chromatographs (Figs. 4c and 5c). This is the first study to prove that *cis*-jasmone and *iso*-OPDA are the origin of pyrethrolone moiety in pyrethrin II. Furthermore, it was found that CJ-*d*_7_ was incorporated without exchanging deuterium protons at C-1 position, while deuterium protons at C-8 position of Me-*iso*-OPDA-*d*_10_ replaced into light hydrogens, which suggested that replacement of protons at the C-2 position of 3,7-didehydroJA was involved in producing pyrethrolone moiety in pyrethrin II (Fig. S11). In the case of applying [U-^13^C] (+)-OPDA, the expected transition ion peaks ascribed for ^13^C labeled JA, JA derivatives (Figs. 6b,c, and S10b), and pyrethrin II (Fig. 7c) were able to be detected, which uncovered that OPDA was used as a biosynthetic intermediate for producing JA and pyrethrolone moiety in pyrethrin II. Based on the above-mentioned experimental results, it was found that the pyrethrolone moiety of pyrethrin II was produced via JA biosynthetic pathway up to OPDA, and then OPDA became a branching point to produce *iso*-OPDA, finally leading the moiety in pyrethrin II. We proposed a biosynthetic pathway to produce pyrethrolone moiety in pyrethrin II via *iso*-OPDA, in which JA was not used as a biosynthetic intermediate (Fig. 8).

Pyrethrins occur in the perennial plant pyrethrum, which has long been cultivated commercially to provide insecticides. Their chemical structures were determined by Hermann Staudinger and Lavoslav Ruzicka in 1924^27^, although the whole picture of biosynthetic pathway of pyrethrins has not been provided. In this report, it was found that OPDA, *iso*-OPDA, and *cis*-jasmone were used to synthesize pyrethrolone moiety in pyrethrin II in pyrethrum, but JA was not. However, some other plant might also make pyrethrin II, and we cannot rule out the possibility that JA could be a source for them. In order to get more detailed information for biosynthetic pathway of pyrethrin, it was thought to be crucial to acquire the information of isomerases that give *iso*-OPDA. To our best knowledge, there are some reports regarding OPDA isomerase, which was obtained from insect guts and thought to be involved in phytohormone detoxification^28–30^.

## Experimental Section

### General

NMR spectra were measured in CDCl_3_ using a JNM-EX 270 FT-NMR spectrometer (JEOL). ^1 ^H NMR spectra was obtained at 500 MHz and ^13 ^C NMR at 125.8 MHz on an AMX 500 (Bruker). A JMS-T100GCV (JEOL) instrument was used for obtaining FDMS and FIMS data. Ultra-performance liquid chromatography (UPLC) was performed on an Waters ACQUITY UPLC system (Waters), and the system was run by MassLynx 4.0. MS/MS was conducted on a Waters Micromass Quattro Premier tandem quadrupole mass spectrometer in multiple reaction monitoring (MRM) mode. Uniform ^13^C-labeled α-LA was purchased from Medical Isotope, Inc. (U.S.A.).

### UPLC MS/MS conditions

Parameters were set according to the reported conditions^31^. UPLC separation was performed on a Waters ACQUITY ethylene-bridged (BEH) C18 column (1.7 μm, 2.1 × 100 mm) at 38 °C. The analytes were eluted from the column with a mixed solvent of 20% aq. MeOH with 0.05% AcOH (solvent A) and MeOH with 0.05% AcOH (solvent B) using a linear gradient program at a flow rate of 0.25 mL/min. To determine JA (method A), 12-OH-JA, and JA-L-Ile, the combination of solvents A and B was 100:0 from 0 s to 0.2 min, and from 0.2 min to 2 min, the combination of A and B was linearly converted from 100:0 to 10:90 and from 8 min to 8.5 min, the combination of A and B was linearly converted from 10:90 to 0:100. The combination of 0:100 was maintained from 8.5 min to 9.5 min. To determine pyrethrins (method B), the combination of A and B was 20:80 from 0 s to 0.2 min, and from 0.2 min to 4 min, the combination of A and B was linearly converted from 20:80 to 0:100. The combination of 0:100 was maintained from 4 min to 7.5 min. The MRM transitions for the tested compounds are given in Tables S1–S4.

### Synthesis of labeled compounds

Synthesis of [^2^H_3_–12, ^2^H_2_–11, ^2^H_1_–10] (±)-MeJA ((±)-MeJA-*d*_6_) and [^2^H_3_–16, ^2^H_2_–15, ^2^H_1_–14] (±)-OPC 6:0 ((±)-OPC 6:0-*d*_6_) were performed according to a previously reported paper^21,24^, [^2^H_2_–4, ^2^H_2_–3, ^2^H_3_–1] *cis*-jasmone (CJ-*d*_7_) and [^2^H_1_–16,^2^H_1_–15,^2^H_2_–11, ^2^H_2_–10, ^2^H_2_–8, ^2^H_2_–2] methyl *iso*-OPDA (methyl *iso*-OPDA-*d*_10_) were performed according to a previously reported papers^19^, although D_2_ gas was used for the dihydrogenation to give methyl *iso*-OPDA-*d*_10_. The synthesis of [U-^13^C] (+)-OPDA was performed according to the report of Kajiwara *et al*.^25^.

### Plant material

Plant materials were prepared according to the reported method^31^ with some modifications. Seeds of pyrethrum (*T. cinerariifolium*) were planted into a Jiffy-7 (ϕ 42 mm) pot, which had absorbed 50 mL of water, and seedlings were grown in the growth chamber. The temperature and moisture of the chamber were set at 25 °C and 60%, respectively, and the conditions of day length were dark for 14 h and light for 10 h. Water was provided once a day, and a 500-fold diluted solution of Hyponex was added once a week as a nutritional supplement. Excess plants were removed in the interim such that there was only one plant per pot. After 35 days, the plants were used in experiments.

### Feeding experiment of airborne (±)-MeJA-*d*_6_

The experiment was performed according to a reported method^23^.

### Feeding experiment of (±)-OPC 6:0-*d*_6_, CJ-*d*_7_, methyl iso-OPDA-*d*_10_, and [U-^13^C] (+)-OPDA and detections of target compounds

Each labeled compound (10 μmol), (±)-OPC 6:0-*d*_6_, CJ-*d*_7_, methyl *iso*-OPDA-*d*_10_, and [U-^13^C] (+)-OPDA, was mixed with approximately 100 mg of anhydrous lanolin. The paste was applied on the surface of the pyrethrum plants. The whole plant material was harvested, frozen using liquid nitrogen, crushed, and soaked with EtOH (20 mL) for 24 h. The extract was filtered, and the volatile components of the extract were removed under reduced pressure. A solution of MeOH (1 mL) was added to the residue, and 2 mL MeOH:H_2_O (80:20) was added to the resulting mixture. The mixture was placed onto a cartridge column of Bond Elut C18 (1 g/6 mL, Agilent). The cartridge was successively washed with solutions of MeOH:H_2_O (90:10) (2 mL × 1) and successively with MeOH:H_2_O (80:20) (2 mL × 2). The volatile components of the combined eluents were removed *in vacuo*, the residue was resuspended in a solution of MeOH:H_2_O (80:20) (0.5 mL), and a portion of the mixture (2–5 μL) was subjected to UPLC-MS/MS. To investigate endogenous JAs and pyrethrins, the methods A and B, which were described in UPLC MS condition of EXPERIMENTAL SECTION, were used, respectively.

## Supplementary information

Supporting information.
