# Supplementary material for: Jasmonic acid is not a biosynthetic intermediate to produce the pyrethrolone moiety in pyrethrin II
Source: Sci Rep. 2020 Apr 14;10:6366. doi: 10.1038/s41598-020-63026-3 (PMC7156398; doi:10.1038/s41598-020-63026-3)
Supplement: Supplementary file 1 — Supporting information. [file 41598_2020_63026_MOESM1_ESM.pdf]

Supplementary Information

Jasmonic acid is not a biosynthetic intermediate to produce the pyrethrolone moiety in pyrethrin II.

Ryo Matsui, Kisumi Takiguchi, Naoshige Kuwata, Katsunari Oki, Kosaku Takahashi, Kazuhiko Matsuda, and Hideyuki Matsuura

From the Laboratory of Natural Product Chemistry, Division of Fundamental AgriScience Research, Research Faculty of Agriculture, Hokkaido University, Sapporo 060-8589, Japan : Graduate School of Agriculture, Faculty of Agriculture, Kinki University, Nakamachi, Nara 631-8505, Japan

List of Supplementary Information

Table S1. UPLC MS/MS parameters for measurements of pyrethrins I and II, jasmolins I and II, and cinerins I and II in positive ionization mode.

Table S2. UPLC MS/MS parameters for measurements of pyrethrin II and its labeled compounds in positive ionization mode.

Table S3. UPLC-MS/MS parameters for measurements of JA, 12-OH-JA and their labeled compounds in negative ionization mode.

Table S4. UPLC MS/MS parameters for measurements of JA-L-Ile and their labeled compounds in negative ionization mode.

Figure S1. Proposed biosynthetic pathway of pyrethrin II.

Figure S2. Reported labeled patterns of *cis*-jasmone derived from [$^2H_1$-7,$^2H_2$-5, $^2H_2$-2] JA (a), and [$^2H_2$-11, $^2H_2$-10, $^2H_2$-8, $^2H_2$-2] methyl *iso*-OPDA (b).

Figure S3. UPLC MS/MS chromatograms for analyzing authentic pyrethrin II (a), jasmolin I (b), and pyrethrin I (c) in positive ionization mode.

Figure S4. UPLC MS/MS chromatograms for analyzing whole plant of pyrethrum (*T. cinerariifolium*) in positive ionization modes.

Figure S5. UPLC MS/MS chromatograms for analyzing authentic JA-L-Ile (a), JA (b), and 12-OH-JA (c) in negative ionization modes.

Figure S6. Feeding experiment using airborne [$^2H_3$-12,$^2H_2$-11,$^2H_1$-10] (±)-MeJA.

Figure S7. Feeding experiment using [$^2H_3$-16,$^2H_2$-15,$^2H_1$-14] (±)-OPC 6:0.

Figure S8. Expected patterns of deuterium labeled pyrethrin II derived from [$^2H_1$-16,$^2H_1$-15,$^2H_2$-11, $^2H_2$-10, $^2H_2$-8, $^2H_2$-2] methyl *iso*-OPDA.

Figure S9. Feeding experiment using deuterium-labeled methyl *iso*-OPDA.

Figure S10. Feeding experiment [U-^{13}C] (+)-OPDA.

Figure S11. Proposed conversion pathway to afford deuterium labeled pyrethrin II from deuterium labeled methyl *iso*-OPDA.

Supporting information Table S1: UPLC MS/MS parameters for measurements of pyrethrins I and II, jasmolins I and II, and cinerins I and II in positive ionization mode.

Target compound	Parent ion (*m/z*)	Transit ion (*m/z*)	Cone Voltage (V)	Collision Energy (eV)
pyrethrin I	330.76	161.54	10	15
pyrethrin II	373.50	161.01	15	12
jasmolin I	332.76	163.54	10	15
jasmolin II [a)]	375.50	163.01	15	12
cinerin I [a)]	317.76	149.54	10	15
cinerin II [a)]	361.50	149.01	15	12

a) parameters were set based on those of authentic pyrethrins I and II, and jasmolin I.

Supporting information Table S2: UPLC MS/MS parameters for measurements of pyrethrin II and its labeled compounds in positive ionization mode.

Target compound	Pseudo molecular ion (*m/z*)	Transit ion (*m/z*)	Cone Voltage (V)	Collision Energy (eV)
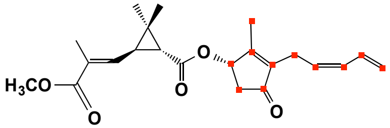	384.50	172.01	15	12
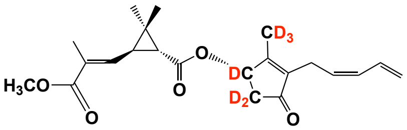	379.50	167.01	15	12
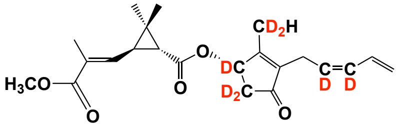	380.50	168.01	15	12
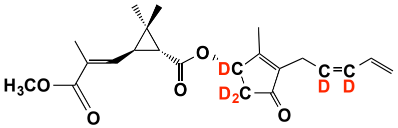	378.50	166.01	15	12
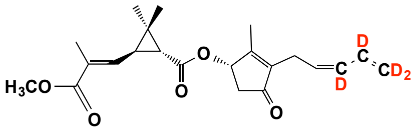	377.50	165.01	15	12
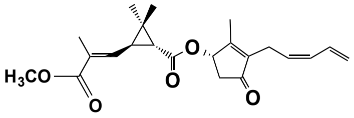	373.50	161.01	15	12

: indicating ^{13}C

Supporting information Table S3: UPLC-MS/MS parameters for measurements of JA, 12-OH-JA and their labeled compounds in negative ionization mode.

Target compound	Pseudo molecular ion (*m/z*)	Transit ion (*m/z*)	Cone Voltage (V)	Collision Energy (eV)
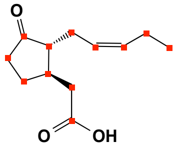	221.00	60.71	24	16
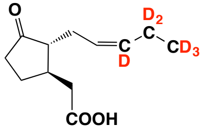	215.00	58.71	24	16
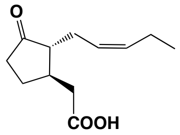	209.00	58.71	24	16
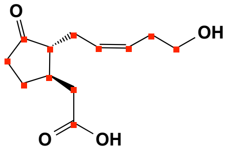	236.97	60.70	32	28
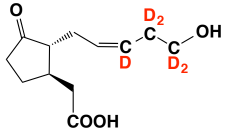	229.97	58.70	32	28
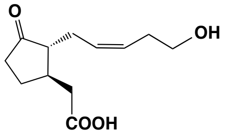	224.97	58.70	32	28

■ : indicating ^{13}C

Supporting information Table S4: UPLC MS/MS parameters for measurements of JA-L-Ile and their labeled compounds in negative ionization mode.

Target compound	Pseudo molecular ion (*m/z*)	Transit ion (*m/z*)	Cone Voltage (V)	Collision Energy (eV)
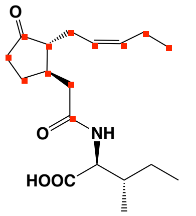	334.03	129.68	45	24
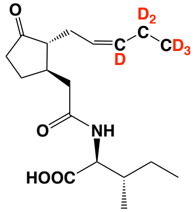	328.03	129.68	45	24
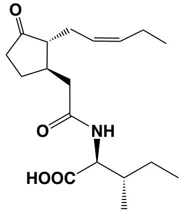	322.03	129.68	45	24

■ : indicating ^{13}C

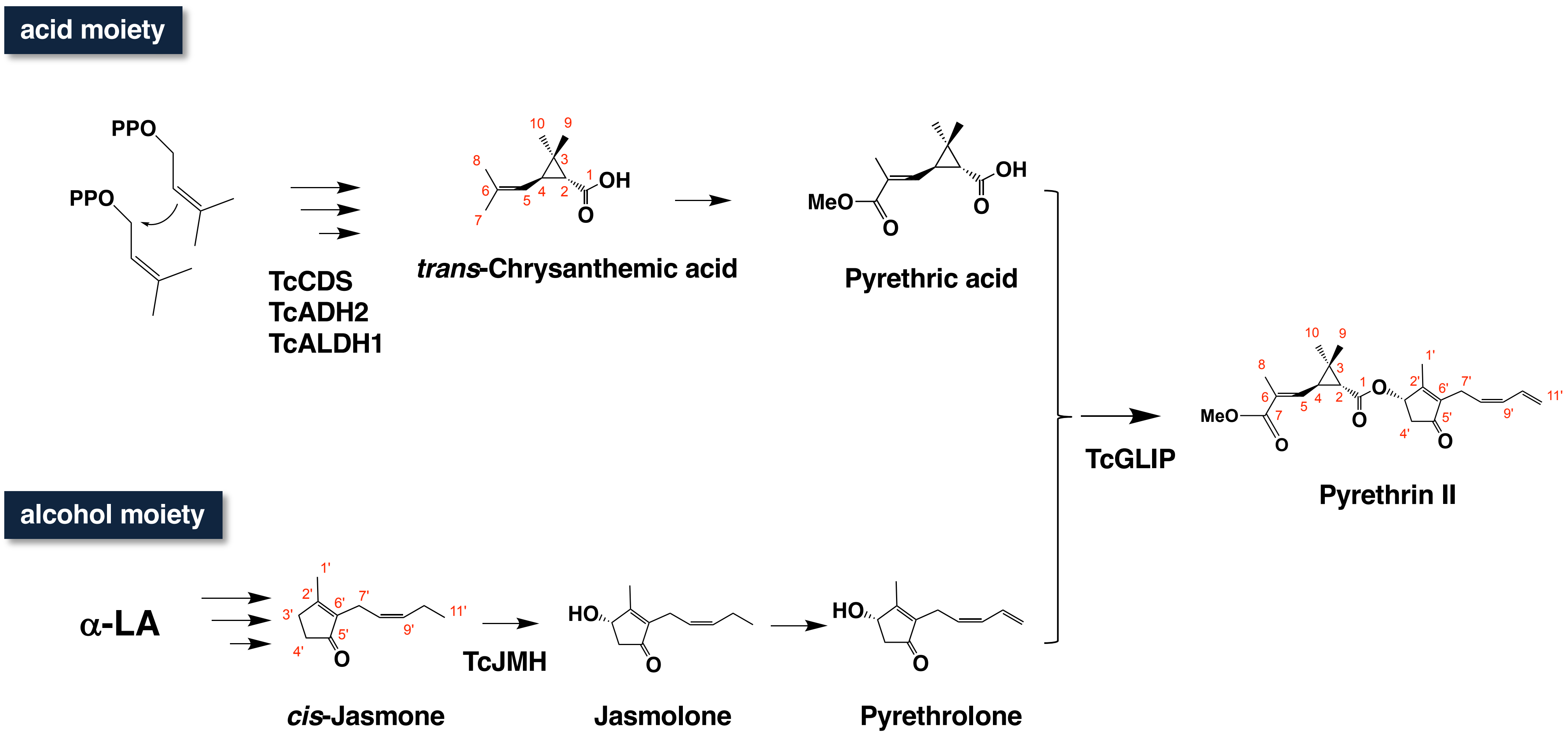

Supporting information Figure S1: Proposed biosynthetic pathway of pyrethrin II.

α–LA: α-linolenic acid, CDS: chrysanthemyl diphosphate synthase, ADH: alcohol dehydrogenase, ALDH: aldehyde dehydrogenases, JMH: jasmone hydroxylase.

a) Koch et. al. (1997) using *Jasrninurn rincospernurn* (1).

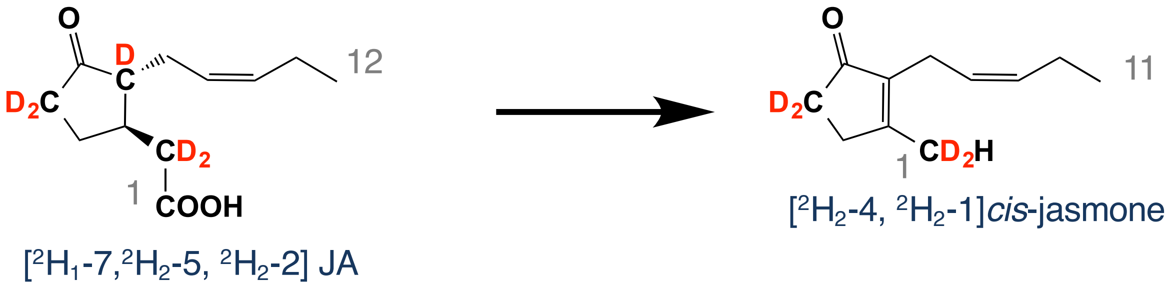

b) Matusi et. al. (2017, 2019) using *Lsiodiplodia theobromae* (2).

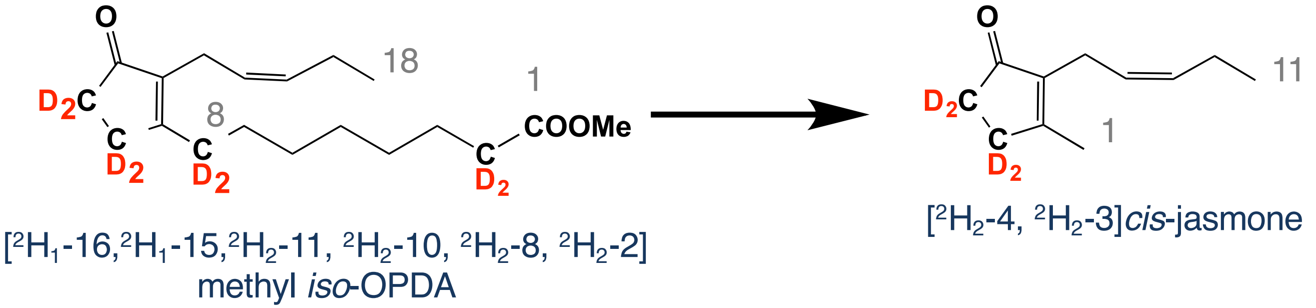

Supporting information Figure S2: Reported labeled patterns of *cis*-jasmone derived from $[^2H_1$-7,$^2H_2$-5, $^2H_2$-2] JA (a), and $[^2H_2$-11, $^2H_2$-10, $^2H_2$-8, $^2H_2$-2] methyl *iso*-OPDA (b).

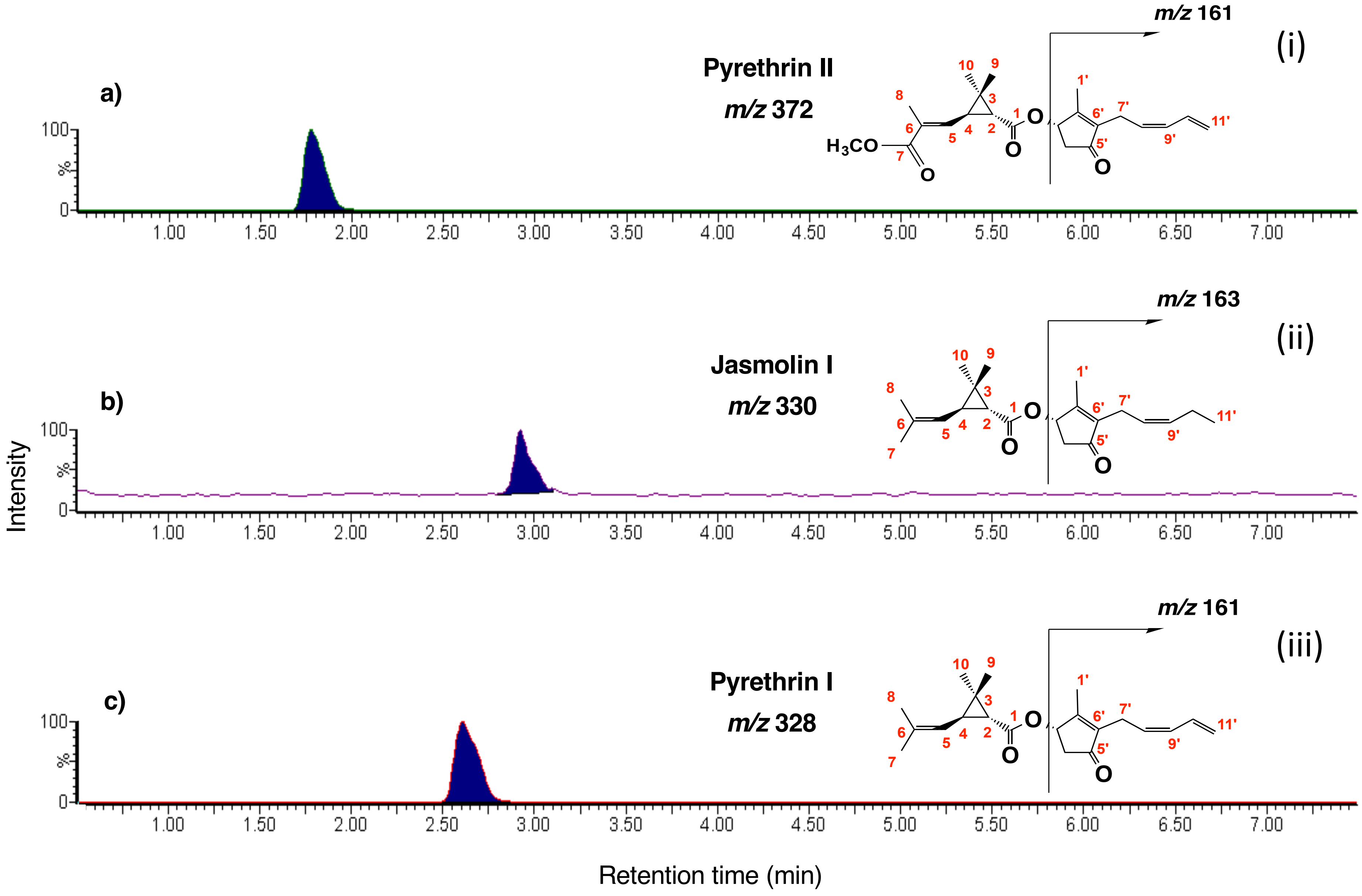

Supporting information Figure S3: UPLC MS/MS chromatograms for analyzing authentic pyrethrin II (a), jasmolin I (b), and pyrethrin I (c) in positive ionization mode. Insets indicate the fragment patterns of pyrethrin II (i), jasmolin I (ii), and pyrethrin I (iii).

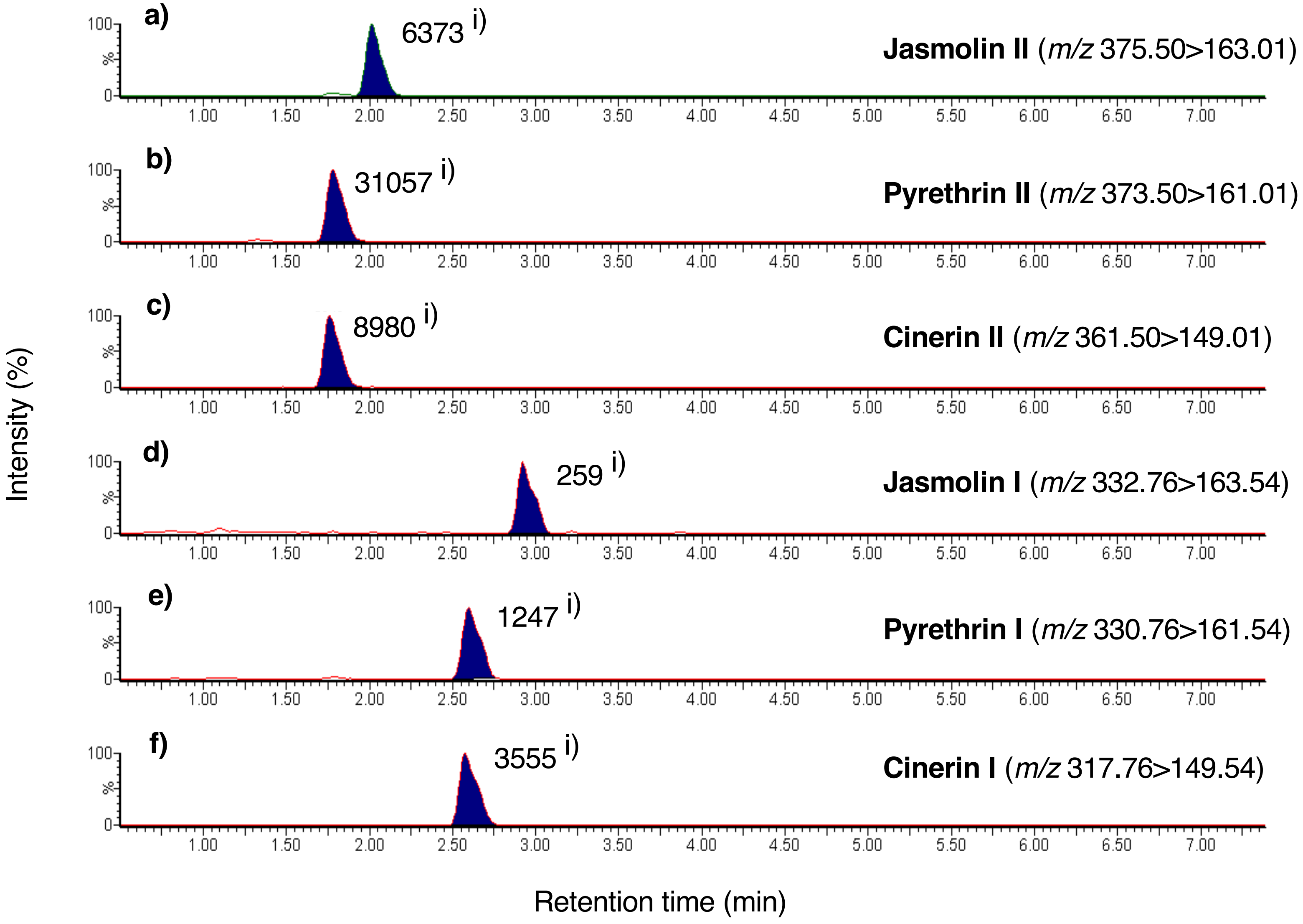

Supporting information Figure S4: UPLC MS/MS chromatograms for analyzing whole plant of pyrethrum (*T. cinerariifolium*) in positive ionization modes. Analyzing jasmolin II (a) , pyrethrin II (b), cinerin II (c), jasmolin I (d) , pyrethrin I (e), cinerin I (f).

i): indicating vale of peak area

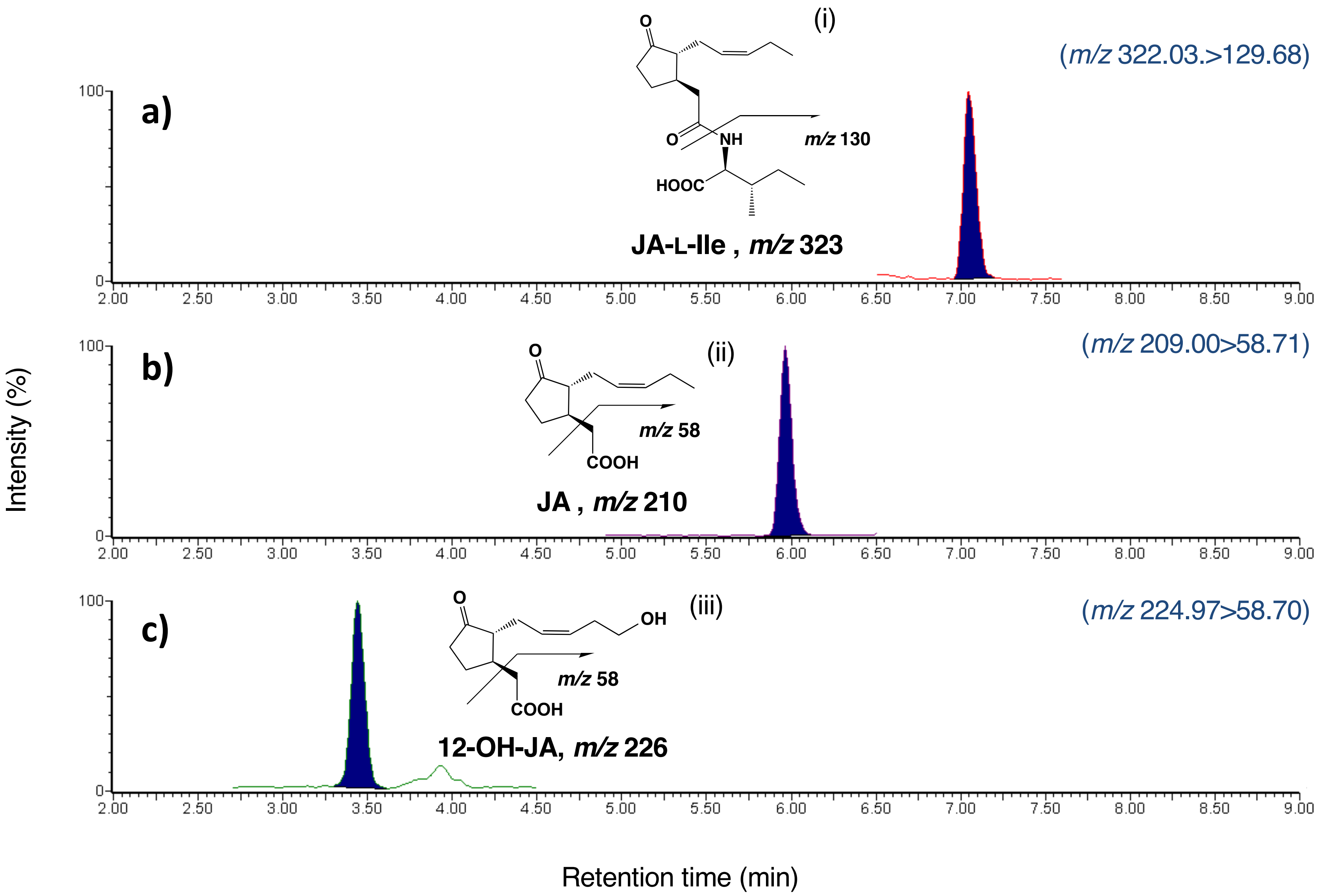

Supporting information Figure S5: UPLC MS/MS chromatograms for analyzing authentic JA-L-Ile (a), JA (b), and 12-OH-JA (c) in negative ionization modes. Insets indicate the fragment patterns of JA-L-Ile (i), JA (ii), and 12-OH-JA (iii).

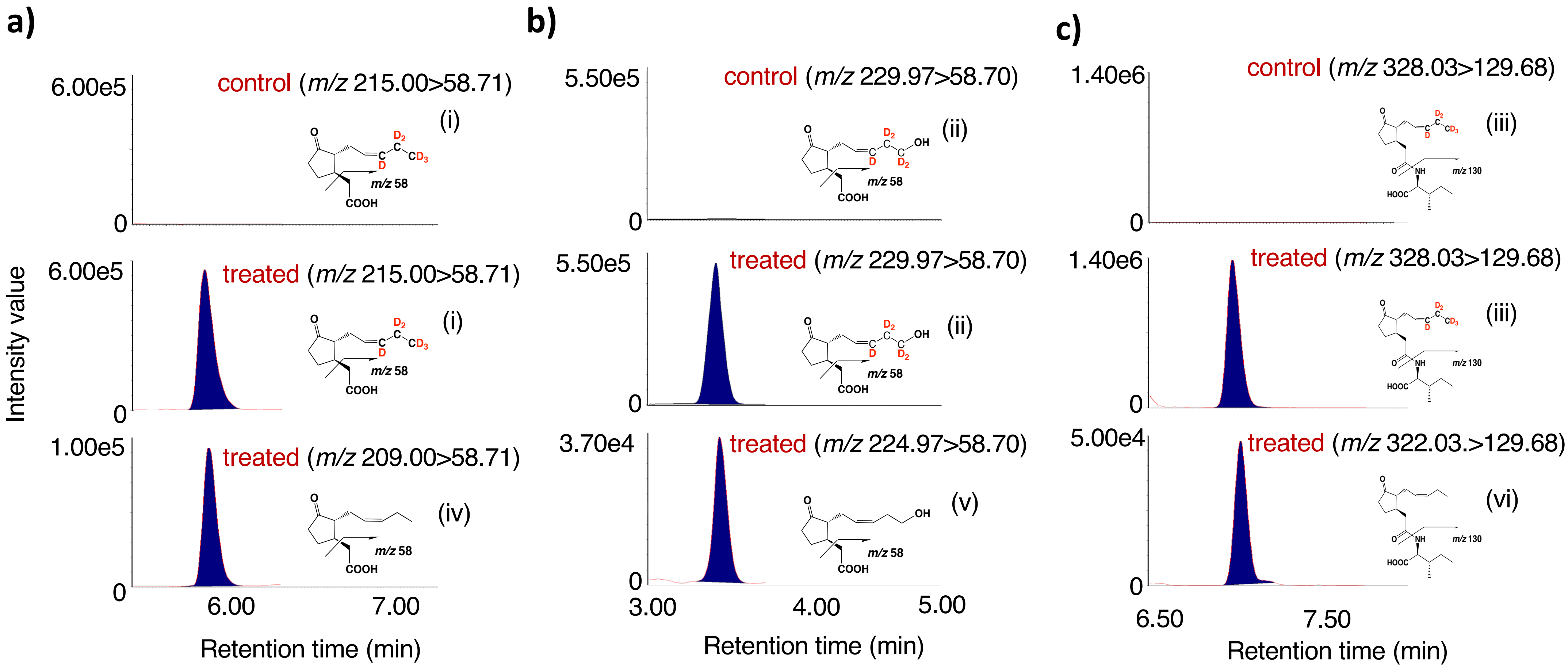

Supporting information Figure S6: Feeding experiment using airborne [$^2H_3$-12,$^2H_2$-11,$^2H_1$-10] (±)-MeJA.

UPLC MS/MS chromatograms of the leaf extract of untreated (control) and treated plants.

Upper and middle panels: JA (a), 12-OH-JA (b), JA-L-Ile (c) having the expected labeled pattern in the control and treated plants, respectively. Lower panel: endogenous target compounds in the treated plants. Insets indicate the fragment patterns of deuterium labeled JA (i), 12-OH-JA (ii),JA-L-Ile (iii) and endogenous JA (iv), 12-OH-JA (v),JA-L-Ile (vi)

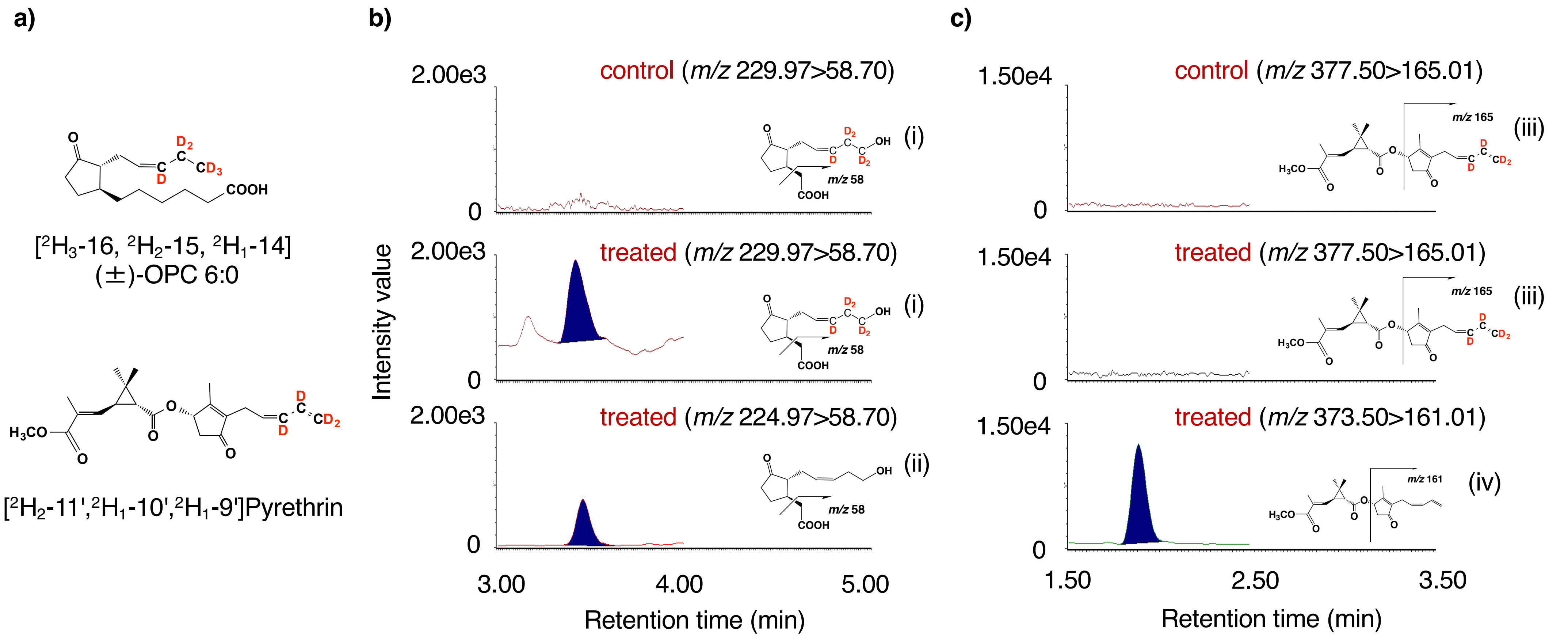

Supporting information Figure S7: Feeding experiment using [$^2H_3$-16,$^2H_2$-15,$^2H_1$-14] (±)-OPC 6:0.

a) Chemical structures of [$^2H_3$-16, $^2H_2$-15, $^2H_1$-14] (±)-OPC 6:0 and pyrethrin II having expected labeled pattern.

b), c) UPLC MS/MS chromatographs analyzing leaf extract of non-treated (control) and treated plants.

Upper and middle panels: analyzing 12-hydroxyJA (b) and pyrethrin II (c) having expected labeled pattern for control and treated plants, respectively. Lower panel: analyzing endogenous 12-hydroxyJA (b) and pyrethrin II (c) for treated plants.

Insets indicate the fragment patterns of deuterium labeled 12-hydroxyJA (i), endogenous 12-hydroxyJA (ii), deuterium labeled pyrethrin II (iii), and endogenous pyrethrin II (iv).

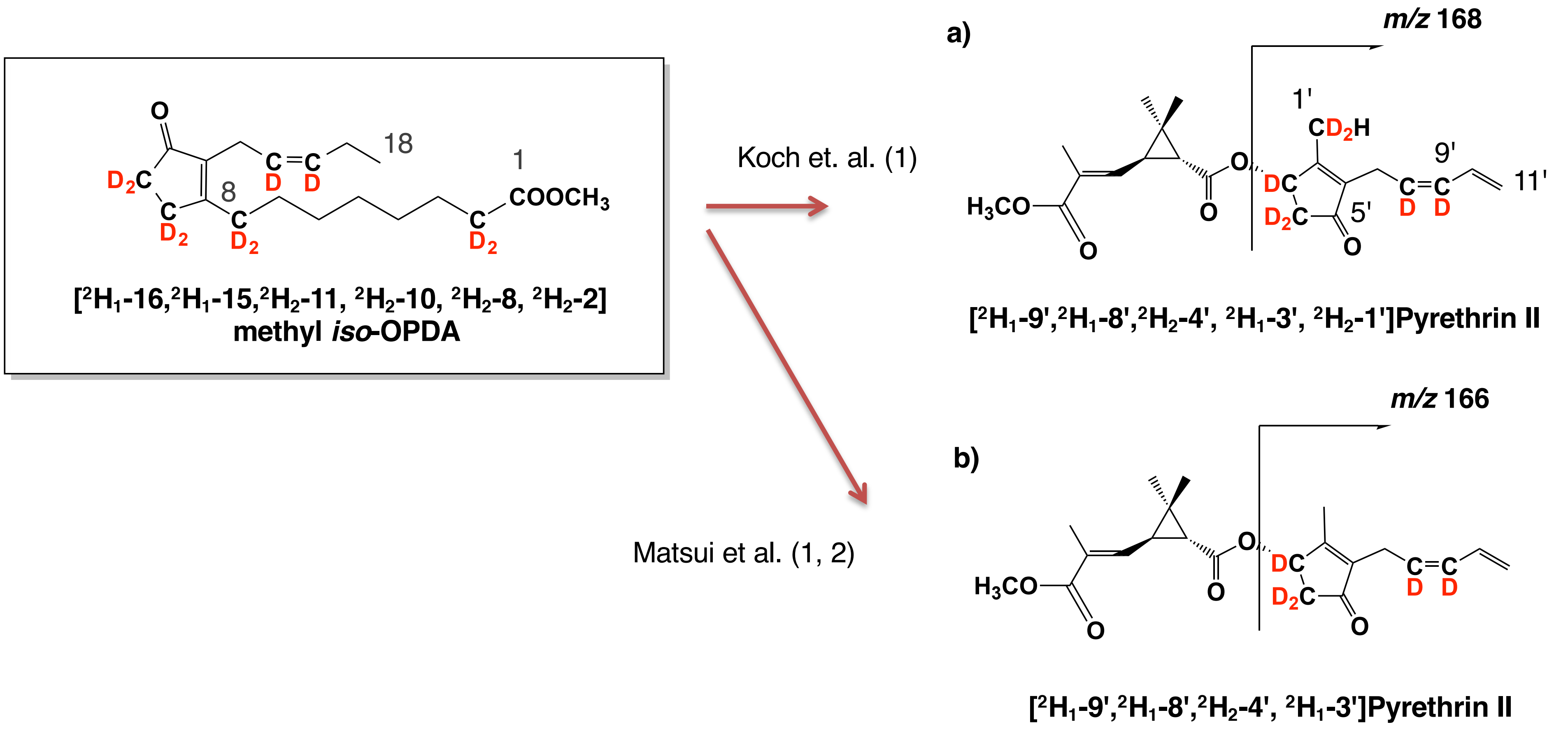

Supporting information Figure S8: Expected patterns of deuterium labeled pyrethrin II derived from [$^2H_1$-16,$^2H_1$-15,$^2H_2$-11, $^2H_2$-10, $^2H_2$-8, $^2H_2$-2] methyl *iso*-OPDA.

The plants were given a lanolin paste having [$^2H_1$-16,$^2H_1$-15,$^2H_2$-11, $^2H_2$-10, $^2H_2$-8, $^2H_2$-2] methyl *iso*-OPDA, and two kinds of labeled patterns were expected. One (a) is based on the report of Koch et al. (1) and the other (b) is Matsui et al. (2, 3).

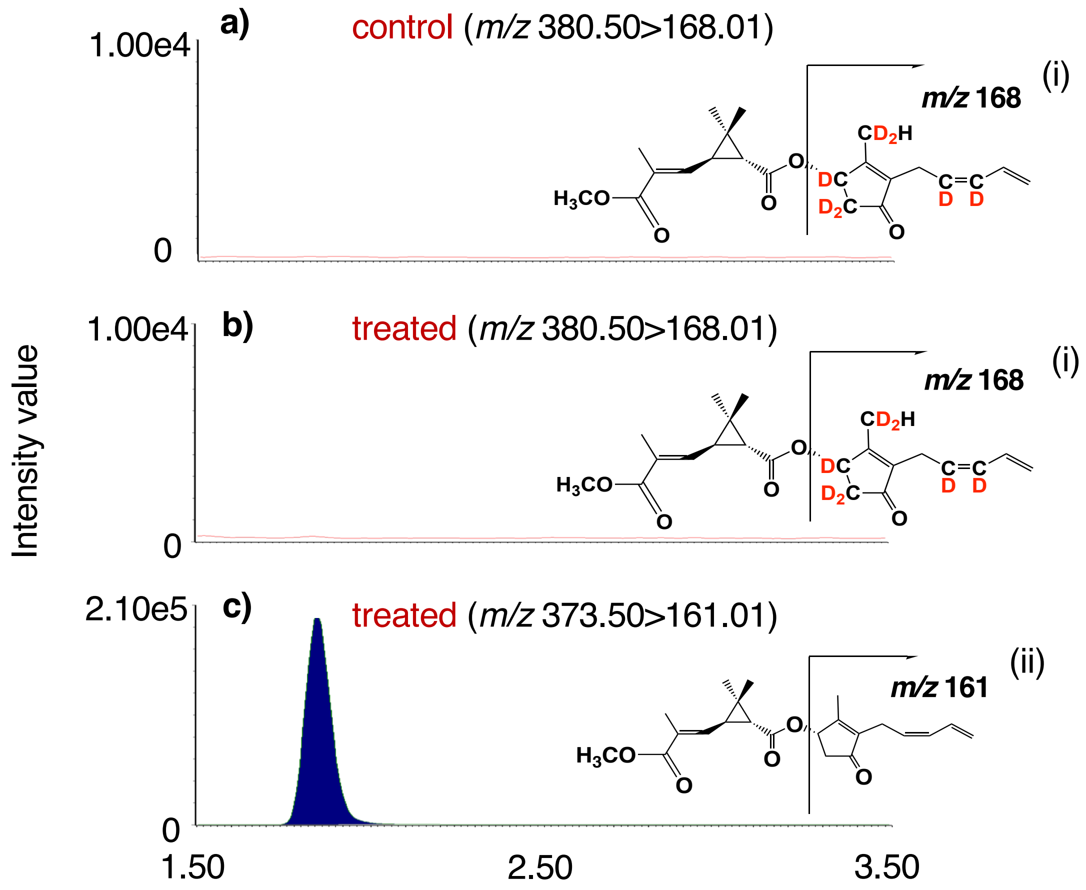

Supporting information Figure S9: Feeding experiment using deuterium-labeled methyl *iso*-OPDA.

a) UPLC MS/MS chromatograph analyzing leaf extract of non-treated plants (control) for [$^2H_1$-9',$^2H_1$-8',$^2H_2$-4', $^2H_1$-3', $^2H_2$-1']pyrethrin II.

b) analyzing leaf extract of treated plant with the labeled compound for [$^2H_1$-9',$^2H_1$-8',$^2H_2$-4', $^2H_1$-3', $^2H_2$-1']pyrethrin II.

c) analyzing leaf extract of treated plants with the labeled compound for endogenous pyrethrin II.

Insets indicate the fragment patterns of [$^2H_1$-9',$^2H_1$-8',$^2H_2$-4', $^2H_1$-3', $^2H_2$-1'] pyrethrin II (i) and endogenous pyrethrin II (ii).

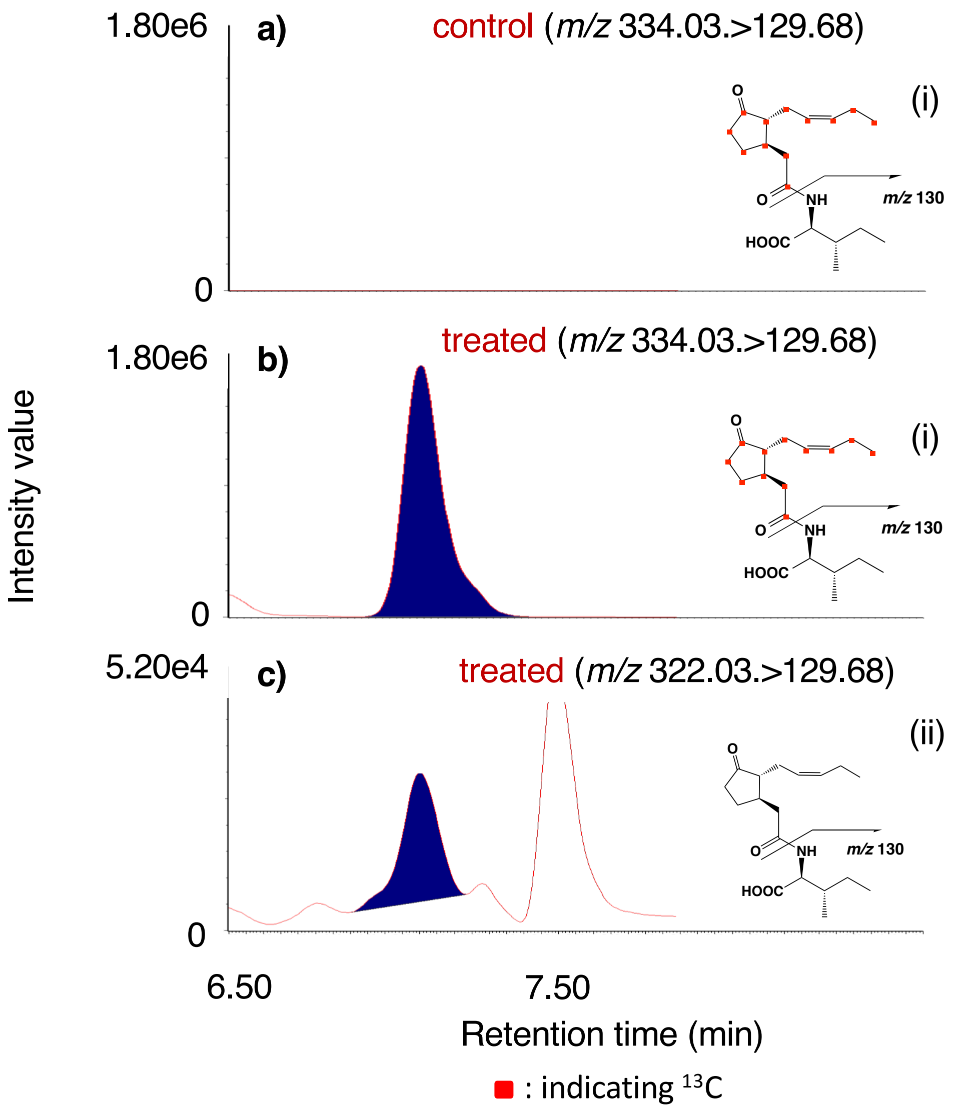

Supporting information Figure S10: Feeding experiment [U-^{13}C] (+)-OPDA.

a) UPLC MS/MS chromatographs analyzing leaf extract of non-treated (control) plant set for [U-^{13}C]JA-L-Ile.

b) analyzing leaf extract of treated plant set for[U-^{13}C]JA-L-Ile.

c) analyzing leaf extract of treated plants set for JA-L-Ile.

Insets indicate the fragment patterns of [U-^{13}C]JA-L-Ile (i) and endogenous JA-L-Ile (ii).

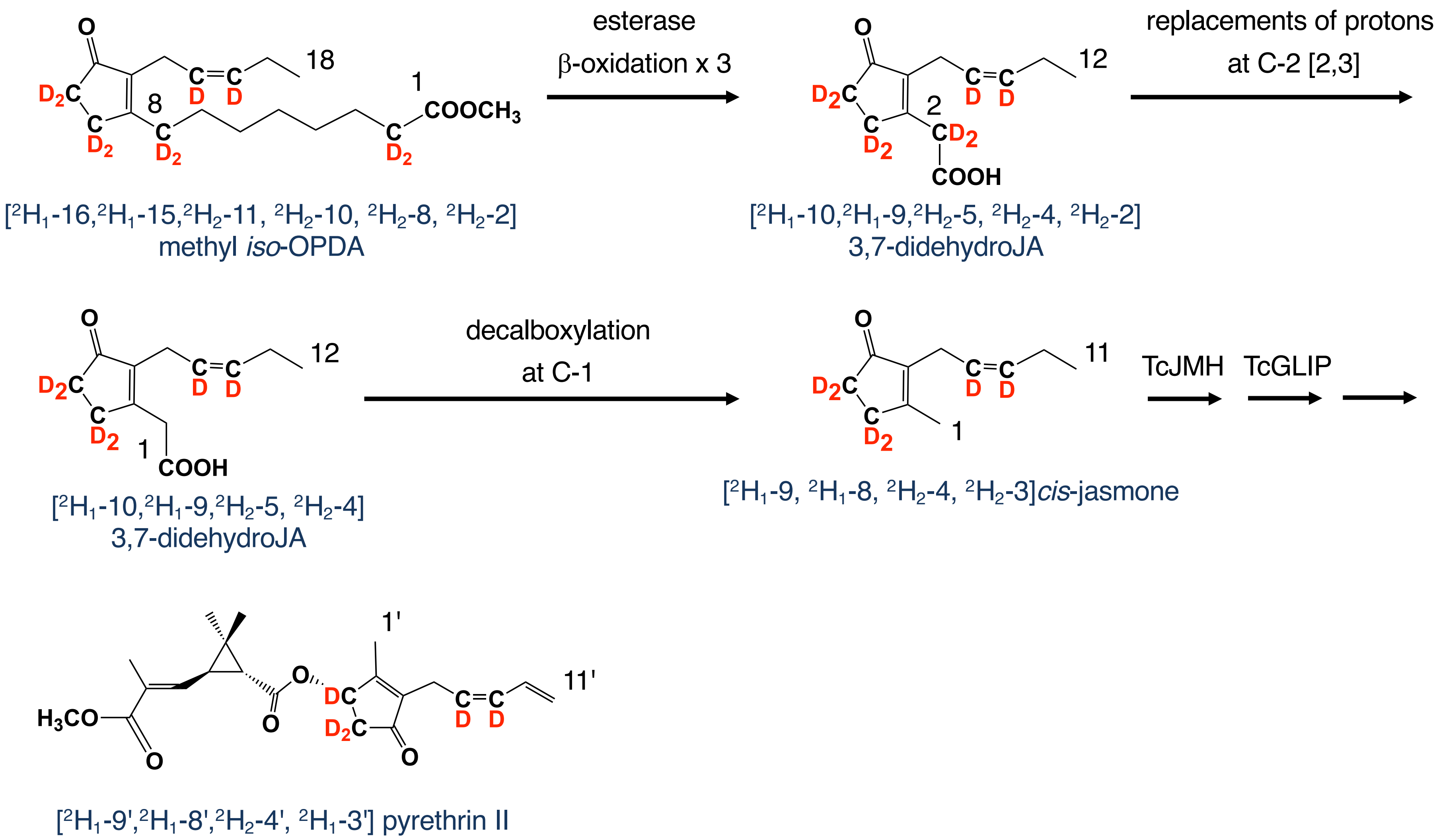

Supporting information Figure S11: Proposed conversion pathway to afford deuterium labeled pyrethrin II from deuterium labeled methyl *iso*-OPDA.

References

1. Koch, T., Bandemer, K., and Boland, W. (1997) Biosynthesis of cis-jasmone: A pathway for the inactivation and the disposal of the plant stress hormone jasmonic acid to the gas phase? *Helvetica Chimica Acta* 80, 838-850
2. Matsui, R., Amano, N., Takahashi, K., Taguchi, Y., Saburi, W., Mori, H., Kondo, N., Matsuda, K., and Matsuura, H. (2017) Elucidation of the biosynthetic pathway of cis-jasmone in Lasiodiplodia theobromae. *Sci Rep* 7
3. Matsui, R., Takiguchi, K., Matsuda, K., Takahashi, K., and Matsuura, H. (2019) Feeding experiment using uniformly (13)C-labeled alpha-linolenic acid supports the involvement of the decarboxylation mechanism to produce cis-jasmone in Lasiodiplodia theobromae. *Biosci Biotechnol Biochem* 83, 2190-2193
